# Increase in early wound leakage in total knee arthroplasty with local infiltrative analgesia (LIA) that includes epinephrine: a retrospective cohort study

**DOI:** 10.1080/17453674.2020.1815975

**Published:** 2020-09-08

**Authors:** Babette C Van Der Zwaard, Ramon L Roerdink, Ruud P Van Hove

**Affiliations:** Department of Orthopedics, Jeroen Bosch Hospital, ’s Hertogenbosch, The Netherlands

## Abstract

Background and purpose — After introducing a new local infiltration anesthesia (LIA) protocol with addition of 30 mL ropivacaine 2% and 1 mg epinephrine, we noted an increase in early wound leakage. As wound leakage is associated with prosthetic joint infection, our department aims to minimize postoperative wound leakage. This study evaluates the incidence of early wound leakage and postoperative pain after knee arthroplasty (KA) following adjustment of the LIA protocol with addition of 30 cc ropivacaine 2% and 1 mg epinephrine.

Patients and methods — In this retrospective medical dossier study all patients (n = 502) undergoing a primary total or unicondylar knee arthroplasty between January 1, 2018 and July 1, 2019 were included. Patients received an LIA protocol containing 120 mL 2 mg/mL ropivacaine (ROPI– group; n = 256). After October 30, patients received an LIA protocol containing 150 mL 2 mg/mL ropivacaine with 1 mg epinephrine in the first 100 mL (ROPI + group; n = 246). The primary outcome measure was early wound leakage (< 72 hours postoperatively), defined as wound fluid leaking past the barrier of the wound dressing. Secondary outcome measure, 10-point numeric rating scale (NRS) pain (< 72 hours postoperatively) was also assessed. Data was evaluated using logistic regression.

Results — The incidence of wound leakage was higher in the ROPI + group: 24% versus 17% in the ROPI– group (p = 0.06). After adjusting for the differences between surgeons the relative risk of this increase was 1.4 (1.0–2.0). The ROPI + and ROPI– group were similar regarding postoperative pain assessment.

Interpretation — Adjustment of the LIA protocol with 30 mL 2% ropivacaine and 1 mg epinephrine led to an increase in early wound leakage in knee arthroplasty but no difference in pain scores.

Local infiltration anesthesia (LIA) during knee arthroplasty (KA) has shown positive results on pain control, early mobilization, hospital discharge, and reduced opiate use (Vendittoli et al. 2006, Toftdahl et al. 2007, Andersen et al. 2008, Gómez-Cardero and Rodríguez-Merchán 2010, Affas et al. 2011).

Several years ago, LIA for KA was introduced in our hospital. Amongst changes in preoperative, peroperative, and postoperative medication, 120 mg of 2 mg/mL ropivacaine was infiltrated in the periarticular tissues during surgery. Pain is considered adequately treated by Dutch quality parameters when the numeral rating score for pain (NRS) is below 4 in 90% of patients during the first 72 hours after surgery (Nederlandse Vereniging voor Anesthesiologie [NVA] 2012). An analysis of our postoperative clinical data showed that this quality parameter could not be achieved with this pain management protocol. Therefore, the LIA protocol was updated to the current Dutch guideline for LIA, in which 150 mL 2 mg/mL ropivacaine with 1 mg of epinephrine was used to infiltrate the periarticular tissues (NVA 2012).

KA can be associated with complications such as wound leakage (Saleh et al. 2002, Patel et al. 2007, Kremers et al. 2019). Early wound leakage, during the first 72 hours after surgery, is generally accepted as normal (Kremers et al. 2019). Prolonged wound leakage can result in delayed wound healing, delayed mobilization, patient dissatisfaction, prolonged hospital stay, and increased costs and has been strongly associated with prosthetic joint infections (PJI) (Kurtz et al. 2008, Bozic et al. 2010, Rietbergen et al. 2016).

After adjustment of the LIA protocol in near conformation with the national guideline, an increase in the number of patients with postoperative wound leakage was observed at our department. The main aim of this retrospective medical dossier study was to evaluate the effect of both LIA protocols on early wound leakage and if a difference was found to assess whether there was a difference in postoperative pain scores between the LIA protocols.

## Patients and methods

### Study design

To evaluate the effect of change in LIA protocol on early wound leakage, this retrospective medical dossier study was conducted. On October 30, 2018 the LIA protocol was adjusted for all knee arthroplasty (KA) patients, which includes both total knee arthroplasties and unicondylar knee arthroplasties. Incidence of early wound leakage was compared for KA patients operated on between January 1 and October 29, 2018 with those operated on between October 30, 2018 and July 1, 2019.

### Participants

Data from primary KA patients operated on between January 1, 2018 and July 1, 2019 were included in the study. Patients operated on before October 30, 2018 were included in the ROPI– group (ROPI–) while those operated on after October 29 were included in the ROPI + group (ROPI+). There were no exclusion criteria. Data was collected from the electronic medical records.

### Oral pain medication protocol

Preoperatively, on the day of surgery, 1,000 mg of acetaminophen, 75 mg of pregabalin, 500 mg naproxen, and 20 mg omeprazol were administered orally. Postoperatively, 1,000 mg of acetaminophen was continued 4 times daily, as well as 75 mg pregabalin twice daily, and 500 mg naproxen twice daily, for 72 hours postoperatively if necessary. Controlled-release oxycodone 5–10 mg twice daily, and immediate release oxycodone 5 mg, 6 times daily if necessary, were also provided for postoperative pain control. Omeprazole 20 mg once daily, granisetron 10 mg thrice daily if necessary and Movicolon (macrogol) 13.8 g once or twice daily were administered to address side effects of pain medication.

### Local infiltration analgesia

In the ROPI– group, 120 mL 2 mg/mL ropivacaine was infiltrated in the periarticular tissues, after the trial components of the total knee prosthesis were removed and before the tourniquet was inflated. The ropivacaine was equally distributed over the posterior and anterior capsule and the subcutaneous tissue around the wound. In the ROPI + group, 100 mL 2 mg/mL ropivacaine with 1 mg epinephrine was infiltrated in the periarticular tissues, as follows: 10 mL for the medial and lateral condyle, 20 mL for medial and lateral posterior capsule, 10 mL on the medial side of the proximal tibia, 10 mL for the patellar ligament, and 10 mL along the distal and proximal quadriceps tendon. 50 mL ropivacaine 2 mg/mL was used to infiltrate the subcutaneous tissue around the wound.

### Surgery

KA was performed either under spinal or general anesthesia by 1 of 5 knee surgeons. Preoperative antibiotics, 2 g cefazoline iv, were administered as well as 8 mg dexamethasone iv and 1,000 mg tranexamic acid iv, prior to the incision. After a medial parapatellar approach, a cemented total knee prosthesis with a fixed bearing (cruciate retained or posterior stabilized, Vanguard, Zimmer Biomet, Warsaw, IN, USA; n = 398 and n = 33 respectively) was placed. In the case of UKA, a cemented unicondylar knee prosthesis with a mobile bearing (Oxford, Zimmer Biomet, Warsaw, IN, USA; n = 71) was placed. A tourniquet was inflated to 250 mm Hg before the components were cemented up to application of the pressure bandage, for approximately 30 minutes. The skin was closed in 4 layers as described elsewhere (Roerdink et al. 2019) After wound closure whilst still in the sterile field the wound was dressed with a 9Ч30 cm Aquacel surgical dressing (Convatec, Greensboro, NC, USA). A pressure bandage was applied for 24 hours.

### Anticoagulants

During hospital stay after surgery, patients were treated with low molecular weight heparin, subcutaneous nadroparine 0.3 mL. Acetylsalicylic acid and carbasalate calcium were only discontinued on the day of surgery and restarted on the first postoperative day. Other antiplatelet therapy and coumarin therapy were discontinued 7 days before surgery and restarted on the 3rd postoperative day only when the wound was dry. Therapeutic direct oral anticoagulants were discontinued.

### Measurements and data sources

The outcome measure is early wound leakage, defined as wound fluid leaking past the barrier of the wound dressing during the first 72 hours after surgery and is defined dichotomously: early wound leakage yes/no. Excessive wound leakage will pass through the sides of the dressing and this is noted in the electronic medical record (EMR). For the search within the EMR for the outcome measure early wound leakage, we used CTCue (CTcue B.V., Amsterdam, https://ctcue.com/). This software can data mine through EMR text and search for keywords regarding wound leakage: wound leaking, wound leakage, leaking wound, dressing saturated, dressing changed. For each of the 3 postoperative days the EMR of each patient was text mined for these keywords. EMRs not containing 1 of the keywords were searched through manually.

Pain was verbally assessed thrice daily by the nursing staff using a numeric rating scale (NRS) ranging from 0 = no pain at all to 10 = most pain imaginable. The results are noted in the EMR of the patient. As NRS pain was a non-normally distributed variable we dichotomized; ≤ 3 and ≥ 4 (van Dijk et al. 2012, Boonstra et al. 2016). Variables such as smoking, diabetes, use of anticoagulants, and surgeon are associated with wound infection. Because early wound leakage might be a symptom of wound infection (Patel et al. 2007, Kremers et al. 2019), these variables were assessed as possible confounders. Smoking, having diabetes, and use of anticoagulants are all assessed during the preoperative screening and recorded in the EMR of each patient.


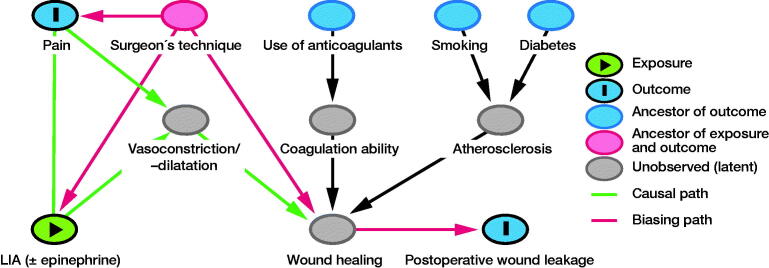


### Study size and statistics

Rule of thumb is to have a minimum of 10–15 cases per variable. An incidence of 24% early wound leakage was found in preliminary data assessment of the ROPI– group. During the inclusion periods 502 patients received a KA. Therefore, this sample had the required power for testing 5 variables, i.e., 1 outcome measure and 4 possible confounders. To evaluate whether type of LIA protocol had an effect on early wound leakage a regression log-binomial model was used. Pain was assessed thrice daily; six consecutive moments of pain assessment were evaluated using generalized estimating equations—a log-binomial model.

The confounder selection was based on the directed acrylic graph (DAG) approach (Shrier and Platt 2008). Possible confounders were selected based on literature on wound infection (Patel et al. 2007, Kremers et al. 2019) and hypothesized relationships. A model of the hypothesized relationships between exposure (type of LIA protocol), primary outcome variable (early wound leakage), and possible confounders (smoking, diabetes, use of coagulants, and surgeon) was created. The final model was chosen after expert-based adjustments and constructed using the online DAGitty (version 3.0) software (Figure) (Textor et al. 2016). Based on the final model, “surgeon” was added to the model as a confounder. 5 orthopedic surgeons performed KA; this ordinal variable was recoded into dummy variables, all of which were evaluated as confounders. If the primary regression coefficient changed > 10% “surgeon” was kept in the final model as confounder. All analyses were performed with SPSS (IBM SPSS Statistics for Windows, Version 25.0; IBM Corp, Armonk, NY, USA).

### Ethics, funding, and potential conflicts of interest

This study lies outside the scope of the Medical Research involving Human Subject Act (WMO), as declared by the Medical Ethical Board Brabant (NW2019-49). The study was not funded by external parties. The authors declare that there are no conflicts of interest regarding the publication of this study.

## Results

### Participants

The cohort consisted of 502 patients, 256 in the ROPI– group and 246 in the ROPI + group (Table 1). The percentage of smokers was significantly higher in the ROPI– group: 14% versus 5% in the ROPI + group (p < 0.001). None of the other baseline characteristics were found to statistically significantly differ between groups.

**Table 1.** Patient characteristics. Values are number (%) unless otherwise specified

	Control group	Study group	
	n = 251	n = 232	p-value
Mean age (SD) **^a^**	69 (8.8)	69 (8.6)	0.5
Female sex **^b^**	154 (61)	134 (58)	0.4
Smoking **^b^**	34 (14)	11 (5)	0.001
Use anticoagulants **^b^**	80 (32)	81 (35)	0.5
Diabetes **^b^**	47 (19)	36 (16)	0.4
Early wound leakage **^b^**	42 (17)	55 (24)	0.06

**^a^** T-test; **^b^** chi-square test.

Direct acyclic graph describing the hypothesized causal pathways of the selected possible confounders.

### Main results

The incidence of wound leakage during the first 72 hours after KA surgery was 17% in the ROPI– group compared with 24% in the ROPI + group (p = 0.06). When the outcome was adjusted for the differences found between surgeons, the RR of this increase was 1.4 (95% CI 1.0–2.0) (Table 2). Neither smoking nor diabetes was found to confound the difference in wound leakage between the groups. As expected, the use of anticoagulants did significantly increase the chance of early wound leakage after KA; RR 1.9 (CI 1.4–2.5). The use of anticoagulants did not, however, confound the difference in wound leakage between the ROPI– and ROPI + group.

**Table 2.** Results of log-binomial models of the ROPI + protocol versus ROPI– protocol

Factor	RR (95% CI)
Early wound leakage—crude model	1.4 (0.99–2.0)
Early wound leakage—adjusted for surgeon	1.4 (1.0–2.0)
NRS pain assessment	0.95 (0.9–1.0)

RR = relative risk; CI = confidence interval;

NRS = numerical rating scale.

Postoperative NRS ≥ 4 were similar between the ROPI + and ROPI– group (Table 2).

## Discussion

On October 30, 2018 the LIA protocol for KA surgeries was adjusted to be in line with the guideline on postoperative pain management from the Dutch Society of Anesthesiology (NVA) because of inadequate pain management (NVA 2012). 30 mL ropivacaine 2 mg/mL and 1 mg epinephrine were added to the existing 120 mL ropivacaine 2 mg/mL. This transition of LIA protocols has led to an increase in the incidence of wound leakage during the first 72 hours after surgery. The protocols did not differ in pain evaluation. The Dutch guideline for LIA after TKA was constructed in 2013 (NVA 2012) and the advice to add 1.5 mg of epinephrine to the 150 mL of ropivacaine was based on expert opinion. When adjusting our LIA protocol, it was hypothesized that the vasoconstrictive effect might inhibit wound healing. Therefore, the new protocol describes the addition of 1 mg epinephrine to the first 100 mL for the periarticular tissue, but not to the 50 mL used for subcutaneous tissue.

In the subsequent years after publication of the guideline, several studies with varying LIA protocols have been published (Karlsen et al. 2017). However, due to the heterogeneity of the protocols, it is not possible to determine an optimal dose and drug regimen based on the current evidence.

Our study is the first to assess the effect of 2 different LIA protocols on early onset wound leakage. We found a higher risk of developing wound leakage during the first 3 days after surgery in the ROPI + group after adjusting for the differences between surgeons. A study comparing the use of ropivacaine with and without epinephrine on postoperative pain perception found “major wound leakage” as adverse event in the epinephrine group (Schotanus et al. 2017). In the epinephrine group 3 out of 25 patients had major wound leakage versus none in the group that received LIA with ropivacaine only. However, the study was underpowered for wound leakage as outcome. It is unclear what was considered “major wound leakage,” but it does seem to corroborate our findings. The causal pathway in which the use of epinephrine could lead to an increased incidence of wound leakage is unclear. One characteristic of epinephrine is that it leads to vasoconstriction. A study on the scalp has shown that the duration of vasoconstriction can vary, depending on the dosage (Na et al. 2016). So one explanation could be that wound leakage increases when the constrictive effect ceases. Another study showed that the incidence of patients with a rise of < 20% of their baseline systolic blood pressure was higher at the moment of release of the tourniquet when epinephrine was used in LIA during KA (Yoo et al. 2020). Although they used a different dosage epinephrine (0.6 mg) and a different consistency of the LIA (ropivacaine 180 mg, morphine sulfate 5 mg, ketorolac 30 mg, cefazolin 1 g, and methylprednisolone 40 mg) (Yoo et al. 2020), the increased blood pressure could account for the increased incidence of wound leakage.

We evaluated 6 consecutive pain assessments with a longitudinal evaluation between the 2 groups and no differences were found between the 2 protocols. Schotanus at al. (2017) found no differences between an 150 mL ropivacaine protocol with or without epinephrine either, which would endorse our findings.

### Limitations

The main limitation of this study is its retrospective nature, in which it is impossible to distinguish between the 2 differences between protocols: the added epinephrine and 30 mL extra ropivacaine. An assumption could be that the extra 30 mL injected LIA could leak out of the wound in its entirety, leading to the increased incidence. In our opinion this is unlikely. The Aquacel surgical bandage used for our wound care is able to absorb 77.5 g/24 hours of wound fluid (1 mL water = 1 g) (absorption information obtained through personal communication with Convatec, C. Lindsay, November 27, 2019). In this study, a surgical incision has to lose more than approximal 77.5 mL/24 hours to be considered as a leaking wound since we defined it as: “wound fluid leaking past the barrier of the wound dressing during the first 72 hours after surgery.” Therefore, it would be likely that the primary reason for our findings is the added epinephrine and not the 30 mL extra ropivacaine.

Another limitation of the study is that we found the surgeon to confound the result. The “surgeon” involves a whole set of small actions and proceedings that might lead to differences between surgeons and it is unclear which of these might lead to differences between surgeons in postoperative wound leakage. This presents an interesting opportunity for future research, but was not feasible to address in the current study. In this study we have done the utmost to evaluate possible confounders. However, due to the retrospective nature of the study and the lack of studies with wound leakage as outcome variables it is feasible that possible confounders have not been assessed or are currently unknown. A clinical relevant outcome measure would be differences in incidence of PJIs between the 2 ROPI protocols. However, the incidence of PJI in this cohort was 0.6% (n = 3), 2 in the ROPI– and 1 in the ROPI + group. This low incidence does not allow any clinical conclusions nor statistical analyses.

## Conclusion

Our results suggest that an LIA protocol of 150 mL ropivacaine with 1 mg epinephrine increases the incidence of early wound leakage after KA compared with a 120 mL ropivacaine protocol without epinephrine. Our findings combined with other studies suggest that the addition of epinephrine to LIA protocols for KA surgeries should be reevaluated.
